# Editorial: 13th international congress on extremophiles: from extremophilic biomolecules and microorganisms to biotechnological and sustainable applications

**DOI:** 10.3389/fmicb.2024.1414760

**Published:** 2024-05-15

**Authors:** Mohea Couturier, Simone Antonio De Rose, Isaac Cann, Melina Kerou

**Affiliations:** ^1^Département de la recherche et de l'innovation, Institut Polytechnique UniLaSalle, Beauvais, France; ^2^The Henry Wellcome Building for Biocatalysis, Biosciences, College of Life and Environmental Sciences, University of Exeter, Exeter, United Kingdom; ^3^Carl R. Woese Institute for Genomic Biology, University of Illinois Urbana–Champaign, Urbana, IL, United States; ^4^Department of Functional and Evolutionary Ecology, Faculty of Life Sciences, University of Vienna, Vienna, Austria

**Keywords:** extremophiles, biotechnology, origin of life, astrobiology, microbiology

Extremophilic environments are routinely exposed to extreme environmental conditions, such as boiling/freezing temperatures, acidic/alkaline pH, high salinity, high pressure, or the presence of toxic substances, where conventional life forms might struggle to survive. These environments are often found in parts of the Earth with inclement conditions, such as deep-sea hydrothermal vents, acidic hot springs, polar ice caps, and hypersaline lakes. Extremophiles are organisms that thrive and adapt to these extreme conditions, demonstrating remarkable resilience and adaptation strategies. These extremophilic microorganisms, based on their living traits, provide insights into the conditions that may have prevailed on early Earth as the planet underwent dramatic environmental changes during its early history. Extremophiles therefore help us understand how life could have emerged and adapted to these extreme conditions. The search for extremophilic niches on Earth, and by extension the study of the associated microbial communities, serve as a model for potential habitats that astrobiology might explore in search for extraterrestrial life signatures. Extremophilic environments are also among the regions most affected by climate change, rendering their study essential in order to understand the impact of changing environmental parameters on microbial communities.

Extremophiles often produce unique biomolecules and enzymes adapted to extreme conditions, called extremolytes and extremozymes, respectively. Extremozymes, for example, can be used in various biocatalytic processes due to their stability under harsh conditions, leading to more efficient and sustainable industrial practices. Indeed, extremophilic enzymes may enhance the efficiency of biofuel production, wastewater treatment, synthesis of pharmaceuticals, and bioremediation efforts to environments contaminated with toxic substances. Enzymes, antimicrobial peptides, and other bioactive compounds from extremophiles may serve as sources for the development of novel pharmaceuticals. Importantly, these molecules may have unique properties that make them effective in combating diseases.

This Research Topic was launched in collaboration with the 13th International Congress on Extremophiles^1^, which took place on September 18–22 in Loutraki, Greece. This International Congress series, supported by the International Society for Extremophiles^2^ aims to promote the latest advances and state-of-the-art research on basic and applied aspects of life in extreme environments. Within this topic, 10 articles have been published not only presenting microbial communities thriving in extremophilic environments but also the biomolecules sustaining such extremophilic lifestyle.

Learning about extremophilic traits starts by characterizing extremophilic environments. The goal is to unravel the role of distinct environmental drivers in such environments which can selectively determine the composition and activity of the resident microbial communities. This Research Topic includes studies presenting the community composition and investigating environmental drivers from radically diverse extremophilic environments such as sub-seafloor hydrothermal sediments of a volcanic field in the Mediterranean sea (Polymenakou et al.) and altitudinal transects of Antarctic valleys (Mashamaite et al.). Polymenakou et al. confirm that high temperature and availability of chemical energy in the form of electron donors and acceptors are major drivers of community structure and diversity, and uncover widespread adaptation strategies to heat stress such as the formation of endospores. Mashamaite et al., 2023 reveal the importance of altitude as a driver of prokaryotic and eukaryotic community structure in Antarctic ice-free soil habitats, resulting in clear distinction between trophic strategies at different altitudes. Among others, an important adaptation and survival strategy for such oligotrophic environments was found to be the capability for oxidation of trace-gases (trace-gas chemotrophy), with the respective taxa being identified as potential keystone taxa for soil communities at higher altitudes.

Taking the reverse approach and studying the distribution, genomic and physiological diversity and potential within a single lineage can also enable us to characterize extremophilic traits. This is the case of the study from Jiang et al., which investigated the distribution of the genus *Solirubrobacter*, showing the enrichment of the genus in arid deserts and high-altitude ecosystems that receive strong solar radiation, but also in the rhizosphere of various plants. Comparative genomic analyses subsequently revealed various molecular mechanisms for counteracting UV radiation, desiccation and osmotic stress, and offer intriguing hypotheses regarding their putatively beneficial role in plant health promotion.

Perhaps the most studied environmental driver for extreme ecosystems is temperature. The report by Lehmann et al. intriguingly challenges the basic assumptions of the study of thermophilic adaptations—that they evolved from a non-thermophilic background. Turning the tables, the study builds upon the widely accepted hypothesis that LUCA may have been a (moderate) thermophile, in which case mesophily would be considered the derived trait. To investigate the molecular evolutionary mechanisms of such a scenario, they subject a thermophilic bacterium with a proposed ancient metabolism (acetogenesis) to adaptive laboratory evolution by continuous cultivation at suboptimal low temperature and map the phenotypic changes that occur.

In addition to physiological investigations, “-omics” approaches can be very useful in extremophilic research in order to provide insights into the molecular mechanisms of adaptations, but their use is often plagued by technical difficulties due to the nature of the samples. Favreau et al. develop methods for -omics analyses of halite samples, and use proteomics to investigate the physiological changes during early acclimation of a model haloarchaeon to halite brine inclusions. Contrary to previous assumptions, the analyzed proteomes of *Halobacterium salinarum* reflect a slowdown in cellular activity and not a stress response during acclimation to the halite environment.

Biochemical characterization of extremophilic enzymes enable us to further deepen our understanding of the molecular mechanisms and trade-offs between stability and activity at extreme conditions, as well as offering candidates for industrial applications. In this Research Topic, the characterization of a novel intracellular subtilisin protease from the bacterium *Planococcus halocryophilus* Or1, metabolically active down to −25°C, is reported by Rasmussen et al., while De Rose et al. report the characterization of a novel cyclic 2,3-diphosphoglycerate synthetase involved in extremolyte production in the archaeon *Methanothermus fervidus*, reflecting the long-standing interest in understanding cellular responses to extreme temperatures. The challenges of overcoming radiation and desiccation stress are addressed by Rollo et al., in a study which aimed to unravel the role of endonuclease III-like enzymes in oxidative stress.

A deeper understanding of the molecular strategies involved in extremophilic adaptations is crucial to addressing societal issues such as toxic compounds contamination. The respective studies of the mechanisms involved in cesium and arsenic resistance offer impressive ways to develop bioremediation projects (Ishida et al. and Gouveia et al. respectively).

## Conclusions

This collection of articles reveals the deep connections between basic and applied research in extremophiles and extremophilic environments. We maintain the notion that the understanding of extremophilic traits is crucial to developing future innovations. Extremophiles offer a rich source of novel biomolecules and enzymes with diverse applications in biotechnology, industrial processes, energy production, and healthcare. By tapping into the capabilities of extremophiles, researchers can contribute to the development of sustainable and innovative solutions that address global challenges and shed light on the process of origin of life on Earth. We anticipate that novel findings and studies, which will be presented during the 14th International Congress on Extremophiles^3^ (September 22-26 2024, Loutraki, Greece), will open the road for future breakthroughs.

## Author contributions

MC: Conceptualization, Investigation, Writing – original draft, Writing – review & editing. SD: Conceptualization, Investigation, Writing – original draft, Writing – review & editing. IC: Writing – original draft, Writing – review & editing. MK: Conceptualization, Investigation, Writing – original draft, Writing – review & editing.

## Footnotes

^1^ https://www.extremophiles2022.org/

^2^ https://extremophiles.org/

^3^ http://www.extremophiles2024.org/

